# Resilient Consensus Control Design for DC Microgrids against False Data Injection Attacks Using a Distributed Bank of Sliding Mode Observers

**DOI:** 10.3390/s22072644

**Published:** 2022-03-30

**Authors:** Yousof Barzegari, Jafar Zarei, Roozbeh Razavi-Far, Mehrdad Saif, Vasile Palade

**Affiliations:** 1 Department of Electrical and Electronics Engineering, Shiraz University of Technology, Shiraz 71557-13876, Iran; y.barzegari@sutech.ac.ir; 2 Department of Electrical and Computer Engineering, University of Windsor, Sunset Ave., Windsor, ON N9B 3P4, Canada; roozbeh@uwindsor.ca (R.R.-F.); msaif@uwindsor.ca (M.S.); 3 Center for Computational Science and Mathematical Modeling, Coventry University, Coventry CV1 5FB, UK

**Keywords:** DC microgrid, attack-resilient control, boost converter, sliding mode observer, false data injection cyber attack

## Abstract

This paper investigates the problem of false data injection attack (FDIA) detection in microgrids. The grid under study is a DC microgrid with distributed boost converters, where the false data are injected into the voltage data so as to investigate the effect of attacks. The proposed algorithm uses a bank of sliding mode observers that estimates the states of the neighbor agents. Each agent estimates the neighboring states and, according to the estimation and communication data, the detection mechanism reveals the presence of FDIA. The proposed control scheme provides resiliency to the system by replacing the conventional consensus rule with attack-resilient ones. In order to evaluate the efficiency of the proposed method, a real-time simulation with eight agents has been performed. Moreover, a verification experimental test with three boost converters has been utilized to confirm the simulation results. It is shown that the proposed algorithm is able to detect FDI attacks and it protects the consensus deviation against FDI attacks.

## 1. Introduction

In recent years, distributed control has received considerable attention due to its high efficiency, simplicity, and reliability. DC microgrids can be represented as a distributed system, and therefore, distributed control techniques are widely utilized to control these systems. However, due to the nature of distributed networks and also advances in cyber attack methods, these systems are vulnerable to malicious attacks. One of the positive points for these systems is the versatility of a wide range of DC sources, which allows these sources to be used simultaneously in a microgrid [1,2,3,4]. Small energy sources such as solar photovoltaics, fuel cells, batteries, and other renewable energy sources (RESes) [3] mainly have low output voltage and need to boost converters to increase the voltage levels up to the network reference. The most popular control techniques used to regulate the voltage are back stepping [5], sliding mode control (SMC) [6,7], model predictive control (MPC) [8,9], and passivity-based control [10]. These methods have the advantages of robustness, stability, optimality, and flexibility [11].

In a microgrid, for supply distributed and different types of loads, we need distributed networked RESes with two features; all must be grid-connected and operate autonomously [12]. In these cases, designing a distributed control law to reach an agreement between all nodes regarding certain constraints that depends on the state of all agents is named a consensus algorithm [13]. Decentralized and distributed controls are two main keys for consensus problems [14].

Decentralized consensus is not vulnerable at a breakdown point, and is considered as a scalable and efficient control for network management [12]. In general, the purpose of distributed control in a DC microgrid is to reach a voltage consensus and proportional current distribution [15]. In the microgrid, each RES is an agent which cooperates and communicates with the neighbors to reach a consensus. Consensus is possible if the cyber networks report the measurements correctly. Any violation of measurement or incorrect reporting will result in an incorrect voltage or current distribution. In communication-based distributed networks, one of the main threats to the network is cyber attacks [16,17,18].

Cyber attacks are very costly for distributed power systems depending on the type, time, and place of occurrence. It causes major economical and technical problems that may be irreparable [19,20]. The most important of these attacks can be categorized into replay attacks, in which transmitted data are stored and repeated periodically, denial of service attacks (DOSA) [21], false data injection attacks (FDIA), and stealth attacks, in which the attackers have sufficient knowledge about the system model, controller, and network architecture [22]. Stealth attacks can easily bypass the bad-data detection mechanisms [19]. These attacks occurs in microgrids by injecting false data to the communication packets over the communication cyber links. Due to the variety of attack categories, denial of service attacks lead to disconnection and are very easy to detect. Therefore, from the viewpoint of attackers, a false data injection attack has a better chance of winning. Therefore, one of the most important attacks is FDIA, which is studied in this article.

There are several data security methods, such as cryptography, user administration, etc.; these are necessary but not enough to protect the systems against all cyber attacks. This is because the attackers are smart and their methods and knowledge about the systems are growing. Prevention is the first step in countering attacks, and the next step is resiliency. In recent years, several strategies have been proposed to detect and reduce the disadvantageous effects of attacks in microgrids. Some approaches are Kalman-filter-based methods to estimate process variables [23], local observers for fault and field level attacks [24], resilient distributed strategies for detecting and isolating time-varying attacks [25], event-triggered strategies proposed to make the control tolerant and robust under DOSA [26], the game theory defense method for a hierarchical networked microgrid structure [27], secure distributed state estimation for the network under FDIA [28], and distributed cyber attack detection for linear large-scale systems by a bank of unknown input observers (UIO) [29]. In general, all these works can be summarized as two main categories, secure robust control, and secure state estimation.

Considering these issues, the main objective of the current work is to design a distributed consensus algorithm to be resilient against false data injection attacks in a DC microgrid. The proposed method is developed based on the distributed observer form of attack detection schemes to achieve a resilient strategy. In this structure, each agent is locally equipped with a detection mechanism and resilient consensus control that utilizes the information of neighbor agents’ states. To regulate the output voltage of each agent, the sliding mode controller is employed while the reference voltage of the controller is generated by the consensus law. Moreover, it is assumed that the converter voltage and current value can be measured. In order to achieve a resilient control structure, the consensus control is designed so as to be resilient in the presence of the FDIAs. Therefore, when an attack is detected, the corresponding agent will be neglected from the agreement process. In order to detect an FDI attack, a bank of sliding mode observers is designed in each agent in such a way that each observer is related to one of the neighbors.

Using the proposed attack detection algorithm, the compliance of received data from the neighboring agents will be checked with the estimated data from the corresponding observer and so the presence of the attack will be detected. The performance of this method has been validated with simulation and experimental tests to conclude that the proposed mechanism is able to detect the presence of attacks effectively and it is resilient. Compared with the existing literature, the main contributions of this article can be summarized as follows.

1. Compared to previous studies such as [12,22], the proposed approach develops a bank of robust observers for each agent that makes the detection and isolation of the false data injection attacks feasible. Therefore, by eliminating the effect of the attack in the consensus law, a resilient control is achieved.
2. The controller and observers are designed based on robust approaches, which is very important in practical applications. It is shown that consensus is successfully achieved even in the presence of cyber attacks, while the modeling uncertainty is considered.
3. A resilient consensus law is proposed to remove the false data injection attacks from the agreement procedure.
4. The practical efficiency of the proposed method is evaluated in an experimental testbed that is close to real-world applications. To this aim, a complex real-time hardware test is performed by MATLAB, Simulink real-time (XPC-Target), LAN communication, FPGA and Microblaze coding, control board design, and three DC-DC boost converters.

The remaining structure of this article is as follows. In Section 2, the basic concepts for graph theory, consensus protocol, and the microgrid model are presented. A model for the communication link attacks is provided in Section 3. Sliding mode controller and observers are designed in Section 4. The proposed resilience consensus law is developed in Section 5. Simulations and experimental validation are presented in Section 6 and Section 7. Finally, the paper is concluded in Section 8.

## 2. Background

### 2.1. Graph Theory

In this section, some basic definitions of graph theory are reviewed. A graph is a set of nodes that are connected to each other by several links. It is noted as G={V,E,A} that represents information flow between the nodes in the network; $V=\{v_{1},\ldots ,v_{n}\}$ is the set of network nodes, where *n* is the number of nodes, E⊆V×V is the set of network links, and $A=[a_{ij}]$ is the adjacency matrix that $a_{ii}=0$, $0<\delta \leq a_{ij}<1$, where δ is a lower bound for gain of adjacency matrix links. If node *i* has access to the states of node *j*, it means there is a link between them, which is denoted by $e_{ij}=\left(v_{i},v_{j}\right)\in E$. The neighbors of node *i* are denoted by $N_{i}=\left\{j\in V:\left(i,j\right)\in E,i\neq j\right\}$, which can communicate with node *i*.

$L={\left[l_{ij}\right]}_{n\times n}\in R^{n\times n}$ is the Laplacian matrix, where $l_{ii}=\sum_{j=1,i\neq j}^{n}a_{ij}$ and $(l_{ij}=-a_{ij}$ for i≠j). The eigenvalues of the Laplacian matrix can be ordered as $\lambda_{1}<\lambda_{2}<\ldots <\lambda_{n}$, where $\lambda_{2}$ is called the algebraic connectivity of the graph. A graph is connected if only its algebraic connectivity is positive: $\lambda_{2}>0$. In a connected graph, agreement will be met, if the condition $\lim_{t\to \infty }\;∥x_{i}\left(t\right)-x_{j}\left(t\right)∥=0,\forall i,j=1,\ldots ,n,$ is established [30,31].

### 2.2. Conventional Consensus Protocol

In a network of agents, reaching an agreement between nodes is called consensus. In general, each node is modeled as

(1) $$\begin{array}{c} x_{i}\left(t\right)=f\left(x_{i}\left(t\right),u_{i}\left(t\right)\right) \end{array}$$

A dynamic graph is shown by (G,x), where *G* is the graph topology and *x* is agents’ states that are described by (1). The consensus problem is described by finding a way to guide agents’ states to an agreement. In a simple and ideal multi-agent system, $u_{i}\left(t\right)$ depends on the states of neighbors that are compared and gained. This is expressed as:

(2) $$\begin{array}{c} u_{i}\left(t\right)=f_{c}\left(x_{i}\left(t\right),x_{j_{1}}\left(t\right),\ldots ,x_{j_{m}}\left(t\right)\right) \end{array}$$

The neighbors of node $v_{i}$ are denoted by $N_{i}=\left\{v_{j}\in V:\left(v_{i},v_{j}\right)\in E\right\}$, in which *m* is the number of neighbors. The consensus protocol is using a function for $u_{i}=f_{c}\left(x_{i},x_{j\in N_{i}}\right)$, which causes asymptotically an stable agreement. It is the main goal in the consensus problems. In general, a consensus rule with a variable topology graph, communication time delay, and asynchronous update for agreement is

(3) $$\begin{array}{c} u_{i}\left(t_{i}\right)=\sum_{v_{j}\in N_{i}}a_{ij}\left(t_{i}\right)\left[x_{j}\left(t_{j}-\tau_{ij}\left(t_{i}\right)\right)-x_{i}\left(t_{i}\right)\right] \end{array}$$

where $a_{ij}\left(t_{i}\right)$ is an entry of the adjacency matrix *A* that may change by time and is related to the edge of $E_{ij}$, $\tau_{ij}<\tau$ is the bounded delay related to the edge $E_{ij}$ at time $t_{i}$, $t_{j}\leq t_{i}$ is the update time for Agent *j*, which shows that the update time for any agent may be different. By (3), each agent state goes to the neighbors’ states and the graph reaches consensus $\lim_{t\to \infty }\sum_{j\in N_{i}}∥x_{i}\left(t\right)-x_{j}\left(t\right)∥=0$.

### 2.3. DC-Microgrid Dynamic

**Figure 1:**
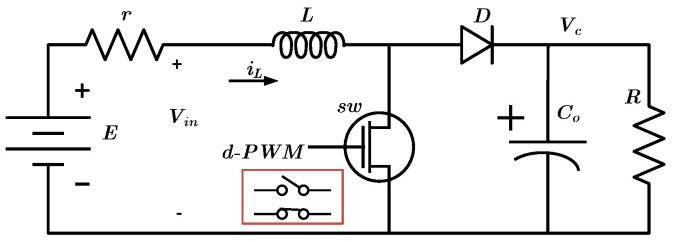
Generalized power model for DC-DC boost converters.

The aim of this section is to introduce a typical model for a DC-DC boost converter in a state space approach. A typical DC-DC boost converter circuit is depicted in Figure 1.

In this figure, $V_{in}$ is the battery voltage, *r* is the sum of inductor resistance and battery resistor, *L* is an inductor, sw is an ideal switch, *D* is an ideal diode, $C_{o}$ is the capacitor, and *R* is the load. The $i_{L}$ is the inductance current that is considered as a state $x_{1}$ and $V_{c}$ is the voltage of the capacitor or output voltage considered as a state $x_{2}$. Based on Kirchhoff’s laws for the ON and OFF states of the switch, two models are given. These two models alternate with switching frequency periods.

Due to the fact that the switching frequency is very high and the rising time and falling time of the switch is very small, the average model for the converter can be used. According to the duty cycle of switch operation (switch is ON for *d* and OFF for (1−d) in any period), the non-linear average model of the DC-DC boost converter is presented in (4) and the linear time-variant state space is presented in (5).

(4) $$\begin{array}{c} \left\{\begin{array}{c} {\dot{x}}_{1}=-\frac{r}{L}x_{1}-\frac{1-d}{L}x_{2}+\frac{1}{L}V_{in}\\ {\dot{x}}_{2}=\frac{-1}{RC_{o}}x_{2}+\frac{1-d}{C_{o}}x_{1}\\ y=x_{2} \end{array}\right. \end{array}$$

(5) $$\begin{array}{c} A_{i}=\left[\begin{array}{cc} \frac{-r}{L} & \frac{-(1-d)}{L}\\ \frac{\left(1-d\right)}{C_{o}} & \frac{-1}{RC_{o}} \end{array}\right],B_{i}=\left[\begin{array}{c} \frac{1}{L}\\ 0 \end{array}\right]\\ C_{i}=\left[\begin{array}{cc} 0 & 1 \end{array}\right] \end{array}$$

where $x\in R^{n}$ is the state vector, $y\in R^{p}$ is the output vector, $u\in R^{q}$ represents the known inputs, and the *r* term is intended to take into account the voltage drop that is caused by the battery current. It is assumed that $A_{i}$, $B_{i}$, and $C_{i}$ are known matrices with appropriate dimensions. For power balance in the steady state, it is:

(6) $$\begin{array}{c} \begin{array}{c} \left\{\begin{array}{c} sw=0\\ Q_{in}=Q_{out} \end{array}\right.\to i_{L}\left(1-d\right)T=I_{out}T\\ \to i_{L}=\frac{I_{out}}{\left(1-d\right)}\to x_{1}=\frac{x_{2}}{R(1-d)} \end{array} \end{array}$$

## 3. Cyber-Physical Attack Model

In the microgrids, two features are very important; first, a global voltage reference exists, which must be followed by all the network nodes, and the second is that all the network nodes must follow the neighboring nodes. With these two requirements, the goal of the network, which is a uniform and homogeneous voltage distribution, is achieved. Because the microgrid consensus is based on interaction and communication within the network, the microgrid consensus is always under threat. Despite all the security and encryption in communications, there are always some attacks aimed at systems by destabilizing goals; thus, the agents must be sensitive to these attacks. In this case, it is assumed that attacks are performed by injecting false data into the output voltage information that transmits between agent neighbors in the network.

**Figure 2:**
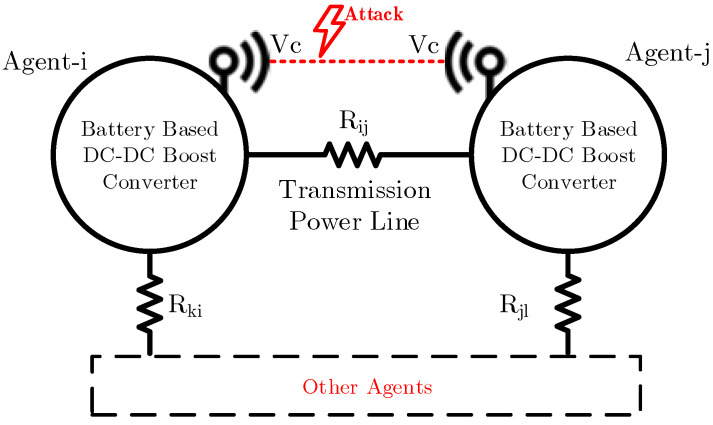
Physical cyber layer and communication link false data injection attack for a DC microgrid.

For cyber-link attacks in the *i*th controller, the attacked value can be modeled as

(7) $$\begin{array}{c} y_{ij}^{at}\left(t\right)=y_{i}\left(t\right)+k_{at}\left(t-T_{at}^{ij}\right)f_{ij}^{at}\left(t\right) \end{array}$$

where $k_{at}$ indicates the attack vector that expresses the existence of an attack, $f_{ij}^{at}\left(t\right)$ denotes the attack function in communication link ij, $T_{at}^{ij}>0$ is the initial time of attack, $y_{i}$ is the real voltage output of Agent *i*, and $y_{ij}^{at}$ represents the attacked value that Agent *j* receives through the communication link about Agent *i*. For example, according to Figure 2, the communication data from Agent *i* to Agent *j* are attacked and the voltage data delivered to Agent *j* are false.

This malicious data lead to an incorrect consensus for the microgrid. For different types of attacks, the $f_{ij}^{at}\left(t\right)$ can take different functions [32]: for FDIA, $f_{ij}^{at}\left(t\right)$ can take any function of time; for a reply attack, it can be $f_{ij}^{at}\left(t\right)=-y_{i}\left(t\right)+y_{i}\left(t-nT\right)$, where *T* is a period of time; in a denial of service attack, it is $f_{ij}^{at}\left(t\right)=-y_{i}\left(t\right)$, which blocks the link by preventing some or all data transmission over the communication link, and for a stealth attack, all data vectors may be replaced with malicious data in such a way that observers cannot find any deviation compared to the system model.

## 4. Observer-Based Attack Detection

To reach a correct consensus in a DC microgrid network, the communication data between the neighboring agents must be correct. If only one piece of the communication data within the network is attacked, the network will reach a false consensus around this value. Therefore, each agent must prevent the influence of defective data. Moreover, because of model parameters’ uncertainty due to factory tolerance, derating, temperature sensitivity, and others, the model is not accurate and control needs to implement a robust strategy. In this paper, a sliding mode controller is proposed to control the converter, and a sliding mode observer (SMO) is proposed to detect the presence of the attack in the received data. To reach consensus in the proposed method, in addition to the conditions mentioned in the graph theory, each agent must be connected to the n+1 neighbors, where *n* is the maximum number of attacks in time. This is because, if *n* attack occurs at the same time, at least a healthy link is needed to achieve consensus.

### 4.1. Sliding Mode Control

The challenge for the boost converter is to design a control law for the duty cycle u(t)∈[0,1] to regulate the output voltage $\lim_{t\to \infty }x_{2}\left(t\right)=x_{2}^{d}$ ($x_{2}^{d}$ is the desired output voltage), while the battery voltage *E* is uncertain and bounded with ΔE<ζ. For this goal, a sliding mode control is designed. The boost converters represent non-linear dynamics with non-minimum phase characteristics [6]. Therefore, the voltage regulation using the switching function $S=x_{2}-x_{2}^{d}$ is not acceptable, though it causes the output voltage to be equal to the desired value. This voltage sliding surface results in an unstable zero dynamic in the inductor current in the sliding motion [33]. According to the relation between position and velocity control, the dynamic of the current is much faster than the output voltage.

**Theorem** **1.** *Consider the system defined in Equation (4). For this system, there exists a distributed sliding mode controller that keeps the microgrid voltages in an asymptotically stable agreement.*

**Proof.**  Design the distributed sliding mode controller for each agent

(8) $$\begin{array}{c} u=u_{eq}+u_{n}=u_{eq}+k\mathrm{sgn}\left(S\right)\\ \mathrm{sgn}\left(S\right)=\left\{\begin{array}{c} 1\\ -1 \end{array}\right.\;\;\begin{array}{c} S>0\\ S<0 \end{array} \end{array}$$

where *S* is the sliding surface that is shown in Equation (9), sgn is the sign function, *k* is the gain for sign function, and $u_{eq}$ is the equal control law that is derived in Equation (11).

To improve the stability of the mentioned sliding mode control, *S* is the state variable trajectory and is described as

(9) $$\begin{array}{c} S=\lambda_{1}{\tilde{x}}_{2}+\lambda_{2}\int {\tilde{x}}_{2}+\lambda_{3}x_{1} \end{array}$$

where the voltage error has been defined as ${\tilde{x}}_{2}=x_{2}-x_{2}^{d}$ and $\lambda_{1},\lambda_{2},\lambda_{3}$ are sliding coefficients. The time derivative of the switching function is

(10) $$\begin{array}{c} \begin{array}{c} \dot{S}=\lambda_{1}{\dot{\tilde{x}}}_{2}+\lambda_{2}{\tilde{x}}_{2}+\lambda_{3}{\dot{x}}_{1}\\ \;=\lambda_{1}\left(\frac{-1}{RC}x_{2}+\frac{1-u}{C}x_{1}-{\dot{x}}_{2}^{d}\right)+\lambda_{2}{\tilde{x}}_{2}+\lambda_{3}\left(-\frac{1-u}{L}x_{2}-\frac{r}{L}x_{1}+\frac{1}{L}E\right)\\ \;=\left(1-u\right)\left(\frac{\lambda_{1}}{C}x_{1}-\frac{\lambda_{3}}{L}x_{2}\right)+\left(-\frac{\lambda_{1}}{RC}x_{2}+\lambda_{2}{\tilde{x}}_{2}-\frac{\lambda_{3}r}{L}x_{1}+\frac{\lambda_{3}}{L}E\right) \end{array} \end{array}$$

where $x_{2}^{d}$ is assumed to be constant, which is calculated by the consensus algorithm in Equation (3). In order to attend to the dynamics of the sliding surface, the time derivative of the sliding surface is investigated. The purpose of this rule is to ensure that, for any initial values, the states will reach the sliding surface. This equation expresses that if we are not on the sliding surface, the path *S* is an absorbing path to the sliding surface. It is found from $\dot{S}=0$ that

(11) $$\begin{array}{c} \begin{array}{c} \left(1-u_{eq}\right)=-\frac{\left(-\frac{\lambda_{1}}{RC}x_{2}+\lambda_{2}{\tilde{x}}_{2}-\frac{\lambda_{3}r}{L}x_{1}+\frac{\lambda_{3}}{L}E\right)}{\left(\frac{\lambda_{1}}{C}x_{1}-\frac{\lambda_{3}}{L}x_{2}\right)}\\ u_{eq}=\frac{\left(\frac{\lambda_{1}}{C}x_{1}-\frac{\lambda_{3}}{L}x_{2}\right)+\left(-\frac{\lambda_{1}}{RC}x_{2}+\lambda_{2}{\tilde{x}}_{2}-\frac{\lambda_{3}r}{L}x_{1}+\frac{\lambda_{3}}{L}E\right)}{\left(\frac{\lambda_{1}}{C}x_{1}-\frac{\lambda_{3}}{L}x_{2}\right)} \end{array} \end{array}$$

The $u_{eq}$ value is calculated for the nominal parameters of the model, and according to the uncertainties of the model, another component must be added to the input to be robust. According to Equation (8), for finding the range of *k* values, the stability condition of the sliding mode controller is $S\dot{S}\leq -\eta \left|S\right|$. For achieving finite-time convergence,

(12) $$\begin{array}{c} S\dot{S}\leq -\eta \left|S\right|,\;\eta >0\to t_{r}=\frac{\left|S(0)\right|}{\eta }\\ \;S\left[\left(-\frac{\lambda_{1}}{RC}x_{2}+\lambda_{2}{\tilde{x}}_{2}-\frac{\lambda_{3}r}{L}x_{1}+\frac{\lambda_{3}}{L}E+\frac{\lambda_{3}}{L}\Delta E\right)+\left(1-u_{eq}-k\mathrm{sgn}\left(S\right)\right)\left(\frac{\lambda_{1}}{C}x_{1}-\frac{\lambda_{3}}{L}x_{2}\right)\right]=\\ S\left[\frac{\lambda_{3}}{L}\Delta E+(-k\mathrm{sgn}\left(S\right)\left(\frac{\lambda_{1}}{C}x_{1}-\frac{\lambda_{3}}{L}x_{2}\right)\right]\leq \\ \left|S\right|\left[\frac{\lambda_{3}}{L}\zeta +-k(\frac{\lambda_{1}}{C}x_{1}-\frac{\lambda_{3}}{L}x_{2})\right]\leq -\eta \left|S\right|\\ \frac{\lambda_{3}}{L}\zeta -k\left(\frac{\lambda_{1}}{C}x_{1}-\frac{\lambda_{3}}{L}x_{2}\right)\leq -\eta \\ k\geq \frac{\eta }{\left|\frac{\lambda_{1}}{C}x_{1}-\frac{\lambda_{3}}{L}x_{2}\right|}+\frac{\lambda_{3}}{L}\zeta \end{array}$$

 □

### 4.2. Sliding Mode Observer Attack Detector

**Figure 3:**
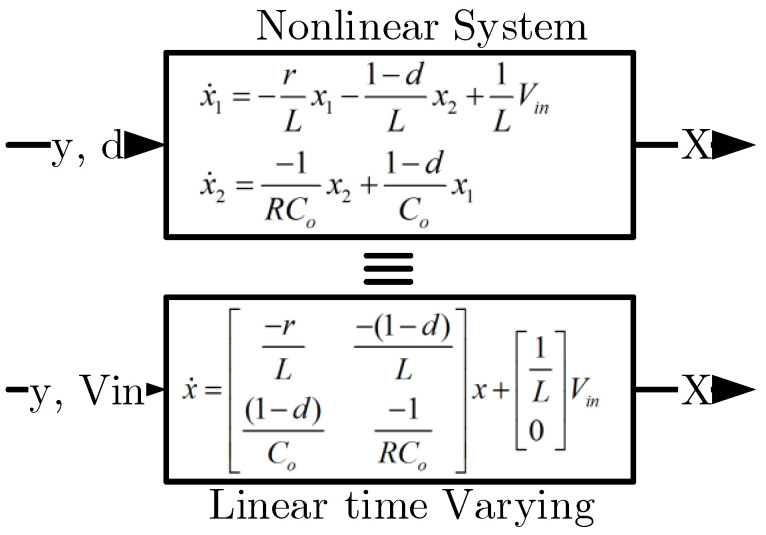
In order to estimate the state vector *X*, two explanations for boost converter modeling from the viewpoint of observer design exist. The first is a non-linear model where *y* and *d* are observer inputs, and the second is a linear time-varying model where *y* and $V_{in}$ are observer inputs.

Observers are dynamic systems that are used to estimate the system states based on the measurements of system inputs and outputs [34]. The estimation occurs when we do not have access to some state variables or we face a fault detection problem. In order to design an observer for the non-linear systems or with parametric uncertainty and perturbation, the sliding mode observers are proposed. It is appropriate for robust estimation, accurate tracking, limited time convergence, and fault detection. In this paper, we convert a non-linear DC-DC boost converter problem to a time-varying linear problem by the assumption that we know the duty cycle values. According to Figure 3, if we have access to the duty cycle *d*, the non-linear model for the boost converter can be replaced with a linear time-varying model. By this definition, nothing changes for the system dynamics, and we can use a linear sliding mode observer for this problem.

In the systems where software controls the process (usually, digital control systems execute some software), the safety of software cannot be measured and proven. In control and automation processes, due to the use of software, one of the approaches that is recommended to increase the safety of the systems is the use of different methods and algorithms for one process to increase the redundancy and security of the system. For this reason, with respect to matters of security and safety, it is recommended to use observers that have a completely different structure from the controllers in order to diagnose attacks and faults; if possible, the implementation methods for controller and observer must be different. The difference in the structure of controller and observer results in the fact that the smallest incompatibilities can be easily detected and catastrophic failures can be prevented.

For the observer, if we consider the system input as *d*, the system is modeled non-linearly, and if we consider the system input as $v_{in}=E$, the system becomes a linear system whose dynamic varies with time. This assumption is correct because the values of these two parameters are always available. Considering these cases, the system state equations can be written as (5).

In order to design an observer, the pairs (A,C) must be observable. Therefore, we form the visibility matrix as

(13) $$\begin{array}{c} Q=\left[\begin{array}{c} C\\ CA \end{array}\right]=\left[\begin{array}{cc} 0 & 1\\ \frac{1-d}{C_{o}} & \frac{-1}{RC_{o}} \end{array}\right] \end{array}$$

If the matrix *Q* is full rank, the system is fully observable. A matrix by dimension of 2×2 has full rank if its determinant is non zero. Thus,

(14) $$\begin{array}{c} \det\left(Q\right)=-\frac{1-d}{C_{o}} \end{array}$$

This value for d≠1 is always the opposite of zero. Given that 0<d<1 (in the simulation and experimental tests in this paper, *d* is about 0.3), this assumption holds. Therefore, the system is completely observable. In the following, we will estimate the system states by using the proposed observer structure.

(15) $$\begin{array}{c} S={\hat{y}}_{ij}-y_{ij}\\ v=sgn(S)\\ G=\left[\begin{array}{c} \beta \\ \gamma \end{array}\right],\left\{\begin{array}{c} \beta \in R^{(np)\times p}\\ \gamma \in R^{p\times p} \end{array}\right.\\ {\dot{\hat{x}}}_{ij}=A_{ij}{\hat{x}}_{ij}+B_{ij}u_{ij}+Gv,\\ {\hat{y}}_{ij}=C_{ij}{\hat{x}}_{ij} \end{array}$$

where *S* is the sliding surface, ${\hat{x}}_{ij}$ is an estimation for $x_{ij}$, $A_{ij},B_{ij},C_{ij}$ are the observer matrix, $u_{ij}$ is the input voltage for the boost converters ($V_{in}$). It describes the input voltage of the ith converter, which is used in the observer of Agent *i*, where this observer is located in Agent *j*, and Gv is a term for robustness. In this problem and for a new matrix definition, we have

(16) $$\begin{array}{c} y=Cx,\;C=\left[0\;1\right]\Rightarrow \;\left[\begin{array}{c} x_{1}\\ x_{2} \end{array}\right]=\left[\begin{array}{c} x_{1}\\ y \end{array}\right] \end{array}$$

and so

(17) $$\begin{array}{c} {\dot{x}}_{i}=A_{i}x_{i}+B_{i}u_{i}\Rightarrow \;\left\{\begin{array}{c} {\dot{x}}_{1}=A_{11}x_{1}+A_{12}y+B_{1}u\\ \dot{y}=A_{21}x_{1}+A_{22}y+B_{2}u \end{array}\right. \end{array}$$

According to (15), for the observer, we have

(18) $$\begin{array}{c} Observer_{ij}:\left\{\begin{array}{c} {\dot{\hat{x}}}_{1_{ij}}=A_{11_{ij}}{\hat{x}}_{1}+A_{12_{ij}}{\hat{y}}_{ij}+B_{1_{ij}}u_{ij}+\beta v\\ {\dot{\hat{y}}}_{ij}=A_{21_{ij}}{\hat{x}}_{1}+A_{22_{ij}}{\hat{y}}_{ij}+B_{2_{ij}}u_{ij}+\gamma v \end{array}\right. \end{array}$$

By calculation of estimation error as follows, we have

(19) $$\begin{array}{c} e\left(t\right)=\left[\begin{array}{c} e_{1}\left(t\right)\\ e_{y}\left(t\right) \end{array}\right]\\ \dot{e}=\left[\begin{array}{c} {\dot{e}}_{1}\\ {\dot{e}}_{y} \end{array}\right]=\left[\begin{array}{c} {\dot{\hat{x}}}_{1_{ij}}-{\dot{x}}_{1}\\ {\dot{\hat{y}}}_{ij}-{\dot{y}}_{ij} \end{array}\right],\;{\dot{y}}_{ij}={\dot{y}}_{i}+k_{at}{\dot{f}}_{ij}^{at}\\ =\left[\begin{array}{c} A_{11_{ij}}{\hat{x}}_{1_{ij}}+A_{12_{ij}}{\hat{y}}_{ij}+B_{1_{ij}}u_{ij}+\beta v\\ A_{21_{ij}}{\hat{x}}_{1_{ij}}+A_{22_{ij}}{\hat{y}}_{ij}+B_{2_{ij}}u_{ij}+\gamma v \end{array}\right]-\\ \left[\begin{array}{c} \left(A_{11_{ij}}x_{1}+A_{12_{ij}}y_{ij}+B_{1_{ij}}u_{ij}\right)\\ \left(A_{21_{ij}}x_{1}+A_{22_{ij}}y_{ij}+B_{2_{ij}}u_{ij}\right)-k_{at}{\dot{f}}_{ij}^{at} \end{array}\right] \end{array}$$

Thus, we have

(20) $$\begin{array}{c} \begin{array}{c} {\dot{e}}_{1}=A_{11_{ij}}e_{1}+A_{12_{ij}}e_{y}+\beta v\\ {\dot{e}}_{y}=A_{21_{ij}}e_{1}+A_{22_{ij}}e_{y}+\gamma v+k_{at}{\dot{f}}_{ij}^{at} \end{array} \end{array}$$

There are two constraints for sliding mode control: first ${\dot{e}}_{y}=0$ to stabilize the error dynamic, and when we are on the sliding surface, $e_{y}=0$ must hold, so:

(21) $$\begin{array}{c} \begin{array}{c} {\dot{e}}_{y}=A_{21_{ij}}e_{1}+A_{22_{ij}}e_{y}+\gamma v+k_{at}{\dot{f}}_{ij}^{at}=0\\ v=\frac{-1}{\gamma }A_{21_{ij}}e_{1}-\frac{k_{at}}{\gamma }{\dot{f}}_{ij}^{at} \end{array} \end{array}$$

and

(22) $$\begin{array}{c} \begin{array}{c} {\dot{e}}_{1}=A_{11_{ij}}e_{1}+\frac{-\beta }{\gamma }A_{21_{ij}}e_{1}-\frac{\beta k_{at}}{\gamma }{\dot{f}}_{ij}^{at}\\ {\dot{e}}_{1}=\left(A_{11_{ij}}-\frac{\beta }{\gamma }A_{21_{ij}}\right)e_{1}-\frac{\beta k_{at}}{\gamma }{\dot{f}}_{ij}^{at}\\ {\dot{e}}_{1}=F_{ij}e_{1}+{\dot{F}}_{ij}^{at} \end{array} \end{array}$$

In order for $e_{1}$ to be stable, $F_{ij}=A_{11_{ij}}-\frac{\beta }{\gamma }A_{21_{ij}}=\frac{-r}{L}-\frac{\beta }{\gamma }\frac{\left(1-d_{i}\right)}{C_{o}}$ must be stable, and then the error tends to zero. Therefore, by selection of β and γ, the error dynamics will be stable. The effect of an attack is $F_{ij}^{at}=-\frac{\beta k_{at}}{\gamma }f_{ij}^{at}$, where the derivation of it appears in the derivation error of state estimation.

## 5. Resilience Consensus Law

In order to achieve consensus when the system is faced with cyber attacks, the consensus law must be revised. The consensus law that is proposed in Equation (3) will be changed to the following equation. The outcomes of the attack observers are now incorporated into the consensus law as a result of this modification. Therefore, the attacked channels will be removed from the consensus protocol.

(23) $$\begin{array}{c} \begin{array}{c} {\tilde{a}}_{ij}=a_{ij}Tr\left(F_{ij}^{at}\right),\;Tr\left(x\right)=\left\{\begin{array}{c} 0\;|x|>threshold\\ 1\;else \end{array}\right.\\ x_{2i}^{d}=u_{i}\left(t_{i}\right)=\sum_{v_{j}\in N_{i}}{\tilde{a}}_{ij}\left(t_{i}\right)\left[x_{j}\left(t_{j}-\tau_{ij}\left(t_{i}\right)\right)-x_{i}\left(t_{i}\right)\right] \end{array} \end{array}$$

where $x_{2i}^{d}$ is the desired output voltage for Agent *i* and Tr(x) is a threshold function.

## 6. Simulation and Results

**Figure 4:**
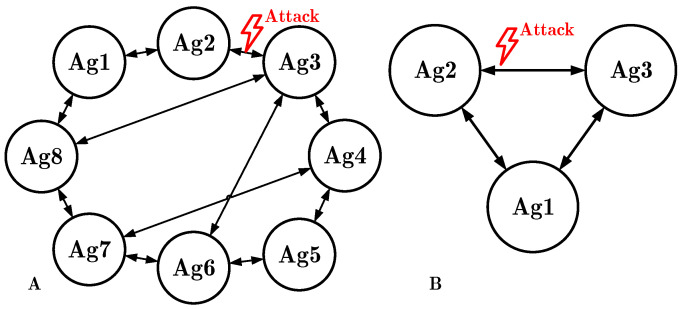
Graph topology: (**A**) eight boost agents for simulation scenario, and (**B**) three boost converters which communicate over the network for experimental test.

In this section, the efficiency of the proposed method has been validated via Simulink real-time (SLRT) simulations. The case study is a network of eight DC-DC boost converters with the non-linear dynamics that are linked as shown in Figure 4A. In this simulation, an attempt is made to choose a graph that considers different modes of connection. In general, the consensus is achieved faster if there are few links between agents; however, it leads to lower reliability as well as more vulnerability to cyber attacks. The coordinating algorithm to achieve consensus becomes more complicated when a large number of communication links is devoted to the agreement process—that is, when the connectivity order of the graph is high, even though it results in greater reliability. Moreover, when the number of participating agents increases, a more complex coordinating algorithm is required. Different components, i.e., sensors and communication links, may be targeted by attackers. The speed of the attack propagation and the scale of the impact will differ; for example, aiming at agents with more connections will result in a faster and greater deviating effect on neighbors. To address this issue, in the proposed algorithm, the communication link that has been attacked is detected, and neglected from the agreement process. On the other hand, aiming at the input communication link of the agent with more neighbors has less effect on the overall graph since it has been removed from the agreement and there are still more inputs to achieve the goal. From a security viewpoint, a large number of connections is desirable because the attack impact is less and the attack is more likely detected. Therefore, for the proposed method, which is based on neglecting malicious input links, a large number of connections is more appropriate. To show the ability of the proposed method, a proper scenario is considered in which agents communicate with a maximum of four neighbor agents.

The goal of this paper is to achieve consensus in the output voltage of decentralized converters in the presence of FDIA. In the simulations, the parameters of converters are E=200 V, r=1Ω, C=2.2 mF, L=2.2 mH, the load resistance R=60Ω and the voltage reference is $V_{ref}=315$ V. The parameters of the sliding mode controller are $\lambda_{1}=1$, $\lambda_{2}=2000$, $\lambda_{3}=0.5$ and the parameters of the observer are γ=1 and the error pole is −600. To draw a comparison between conventional controllers and the proposed algorithm, two simulation scenarios are performed as follows.

**Figure 5:**
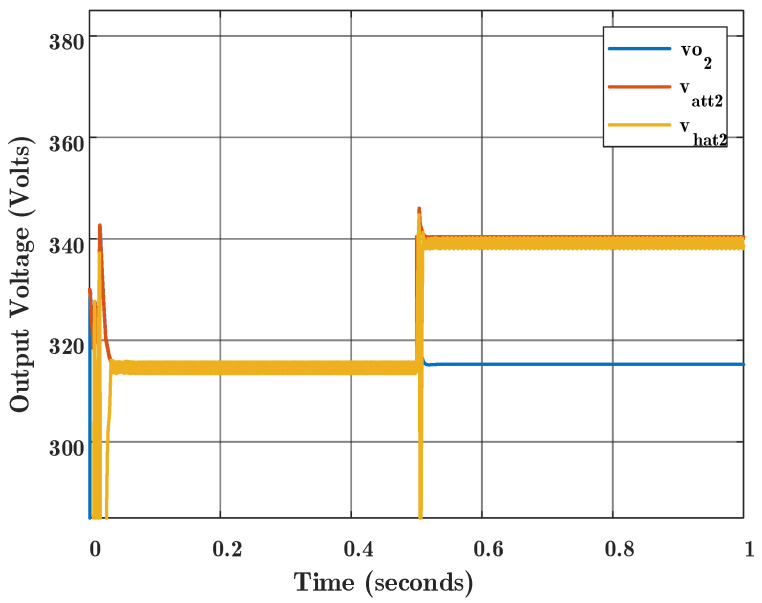
Voltage values for Agent 2, and the concept for what happens when an attack occurs; $Vo_{2}$ is the real output voltage for Agent 2, $V_{att2}$ is the information about voltage of Agent 2 that is delivered over the communication link to Agent 3, which is manipulated by the false data injection attack, $V_{hat2}$ is the voltage observer for Agent 2 that is located in Agent 3.

**First scenario:** All the agents and communication links are healthy. The communication links are with [1, 2, 3, 1, 1, 2, 1, 2] sample delay and they are synchronous. In the time stamp of 0.5 s, an FDIA occurs over the communication link from Agent 2 to Agent 3. As shown in Figure 5, the output voltage of Agent 2 which is delivered to Agent 3 is different from the real output voltage of Agent 2 due to a cyber attack that injects a false datum into the communication link $L_{23}$. Thus, the observer which is located in Agent 3, and estimates the states of Agent 2, follows the attacked voltage, which is different from the real output voltage of Agent 2. Figure 5 is shown for a better understanding of what is happening. This figure explains the attack effect on the communication data, which shows that when the output voltage of Agent 2 is at the steady state (blue color), an attack occurs at 0.5 s and the reported voltage over the communication link deviates from the output voltage of Agent 2 (red color). Therefore, the observer of Agent 2 that is located in Agent 3 follows the attacked value.

**Figure 6:**
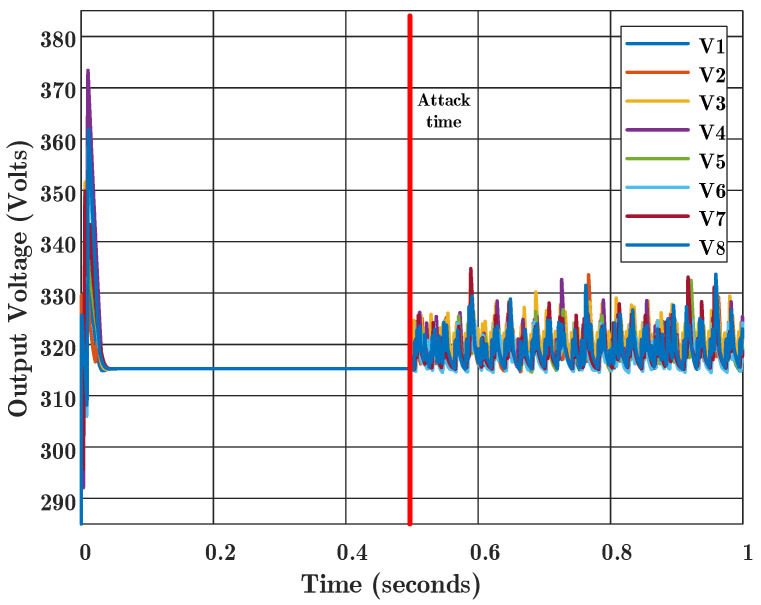
Output voltages of boost converters for conventional consensus algorithm in the presence of false data injection attack at time 0.5 s. The values of $V_{1}$ to $V_{8}$ are output voltages for Agent 1 to 8, respectively.

As shown in Figure 6, by the conventional consensus control law, agreement deviates from the normal condition and the FDIA cyber attack is successful. According to this figure, the output voltages of all agents will deviate because all of them are connected to each other by the communication links. In this scenario, when Agent 3 receives the wrong data, the controller regulates its output voltage to a false value, and this false value is sent to the other agents over the communication links.

**Figure 7:**
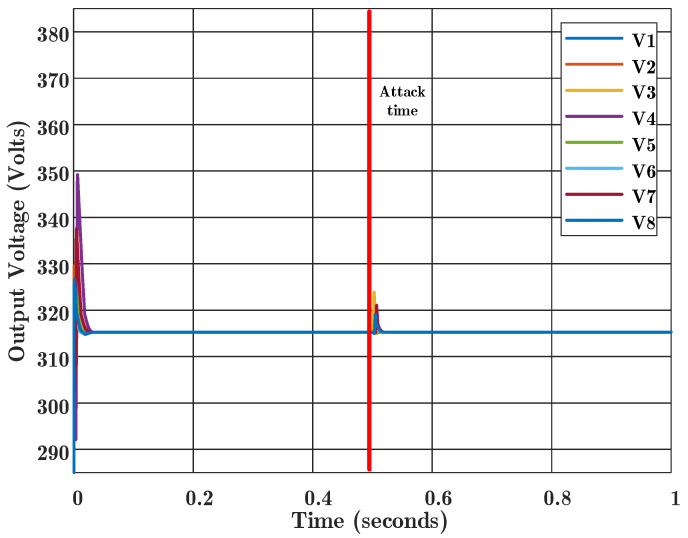
Proposed consensus algorithm results in the presence of false data injection attack at time 0.5 s. The values of $V_{1}$ to $V_{8}$ are output voltages for Agent 1 to 8, respectively.

**Second scenario:** This scenario is the same as the first one, except that the consensus algorithm that is used to detect the attack is based on the developed algorithm in this paper. As shown in Figure 7, the voltages will reach consensus again immediately after the attack has occurred. Therefore, according to Figure 7, it is clear that the consensus will not deviate from FDIA cyber attacks and the consensus process will be performed properly in the presence of this type of attack. As shown in this figure, the attack at Agent 3 affects the other agents. The proposed algorithm detects the source of the attack using residuals from the observer banks, and removes the attacked communication links from the consensus process. Therefore, it is shown that the proposed algorithm has resiliency or attack-tolerant control abilities.

## 7. Experimental Results

**Figure 8:**
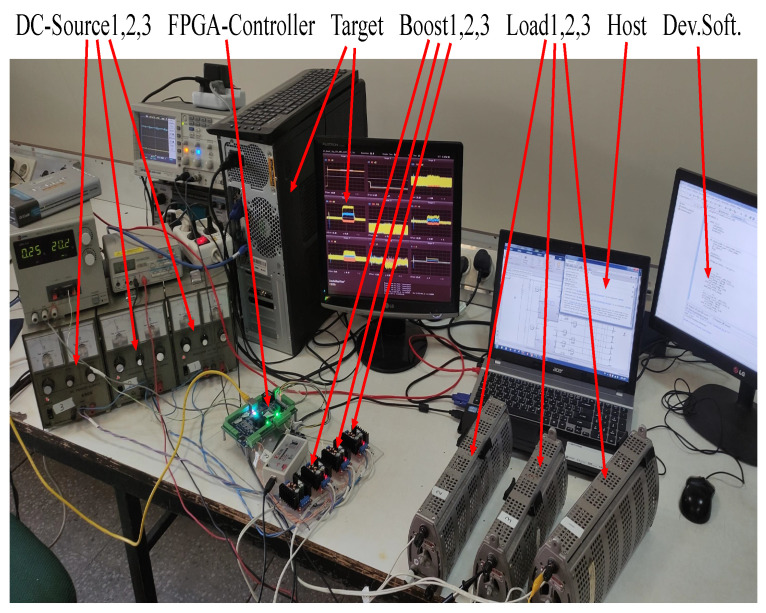
Experimental prototype for the validation of the proposed algorithm. This test-bench is composed of: development computer; host computer; target computer; Spartan 6 FPGA controller; three boost converters; three power supplies; three transmission loads, and a LAN switch.

In order to validate the results, an experimental prototype with three agents is prepared according to the graph shown in Figure 4B and the hardware shown in Figure 8. Due to limited laboratory equipment, the number of agents is reduced to three, and the main reason is that the control board does not support more than three channels. However, a different control board with more channels can be utilized for practical implementation. Moreover, due to the fact that the laboratory power supplies have limited output voltages which are less than 30 volts, the operating voltage is reduced. However, it is worth noting that the nature of the experimental test is not different from the simulations.

This test-bench consists of: a development computer for FPGA and Microblaze programming by Xilinx-ISE and Xilinx-SDK softwares with a JTAG Xilinx programmer; a host computer to generate MATLAB codes, boot the target computer over the network, set-up and logging data from the target computer; a target computer that is booted by the Simulink real-time kernel and runs the tolerant consensus algorithm in real-time and communicates (Ethernet-UDP) with FPGA; a Spartan 6-based FPGA control board that is a controller and logger for the boost converters in an independent and very fast structure; three boost converters that are placed at the graph nodes and supply the hmic loads; three boost power supplies to supply the converters; three ohmic loads for three agents; three transmission ohmic loads between the agents to simulate the transmission power losses and a 100 Mbps Ethernet switch for connection between agents, host computer, and the target computer. In general, this testbed consists of three boost converters that are tied in a physical ring-bus network and a communication network. In order to implement three independent control loops for three agents, an FPGA Spartan 6 based board is used. This processor is connected to the target computer via a LAN-UDP connection link. For ease of programming and debugging, some local control loops are implemented in the Microblaze Xilinx-SDK environment. Boost converters are a 150 watt commercial type with a maximum operating voltage of 36 volts. Each agent consists of an ohmic load of 23 Ω, a boost converter with an efficiency of about 90%, a power supply with a voltage output about 17 volts, and 5 Ω transmission lines. The consensus control algorithm is implemented in the MATLAB software using Simulink real-time. Simulink and FPGA data are exchanging via the LAN connection link with 1 and 10 kHz times updates.

**Figure 9:**
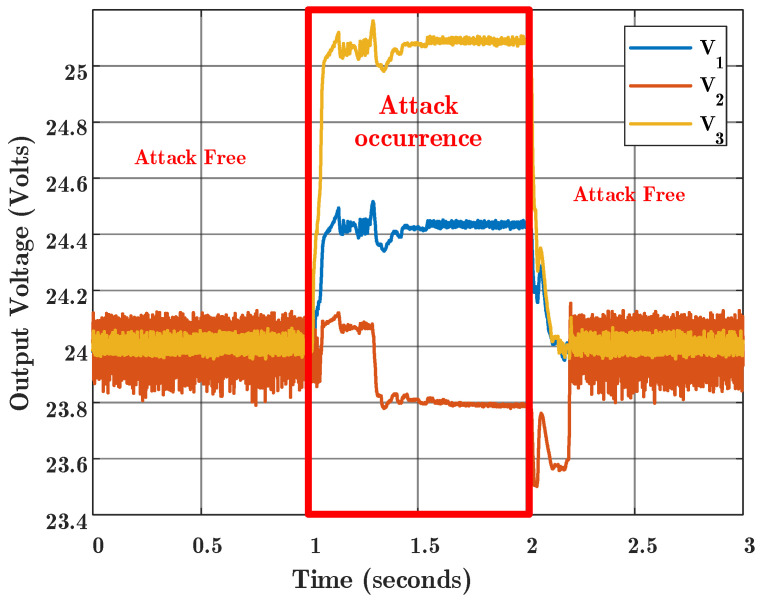
Experimental results for conventional consensus algorithm. FDI attack occurred at time 1 s and was removed at time 2 s.

According to Figure 9, the false data injection attack is aimed at the communication link $L_{23}$ between t=1 and t=2 s. It is shown that for the conventional consensus algorithm, the output voltages of the converters deviate and consequently the consensus mechanism also is violated. Therefore, the conventional consensus algorithms are vulnerable in the presence of attacks. The attack occurs by injection of a fast ramp voltage from 24 to 28 volts into the $L_{23}$ communication channel. Due to the fact that the converters are connected to each other through the 5 Ω power transmission lines, in practical applications, and in this experimental test, the output voltage measurement for each agent is affected by the other agents, and the voltage distribution is not ideally distorted. According to this figure, the maximum deviation is related to Agent 3, which is directly attacked. It is observed that after the end of the attack time, the consensus returned to its normal behavior.

**Figure 10:**
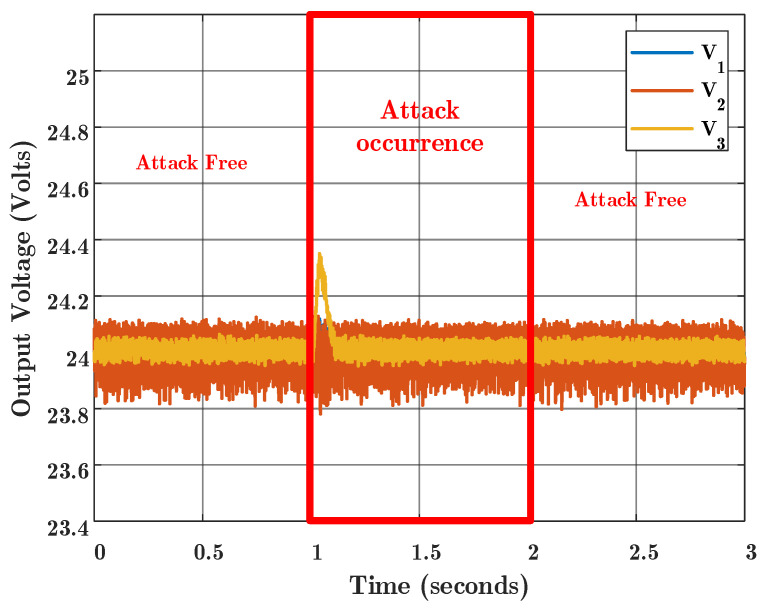
Experimental result for proposed consensus algorithm. FDI attack occurred at time 1 s and was removed at time 2 s.

Figure 10 also shows that with the proposed algorithm, the effect of the attack is eliminated and the consensus for the graph will occur correctly. This figure shows that the proposed tolerant consensus is resilient in the presence of the FDI attacks. It is shown that using the proposed algorithm after the attack has occurred, the attack is successfully detected and isolated, and then the attacked channel is removed from the consensus process to achieve the agreement. This amount of deviation at the start of the attack is shown in Figure 10, which is actually due to the fact that the detection process and the control loops are running in parallel. The cost that this method imposes on the system is the requirement of a larger computational burden compared to the conventional method, and also this method needs to know the model of each agent. It is worth noting that these costs are not comparable with the damages that may result due to cyber attacks.

## 8. Conclusions

In this study, an observer-based resilient control method was proposed to reach the consensus in a DC microgrid. In this microgrid, each agent is a battery-based boost converter and, at an unknown time, a false data injection attack appears. In order to control the voltage for each agent, the sliding mode control method has been used. To estimate the states of the neighboring agents, a bank of sliding mode observers has been proposed, which is organized to detect the attacks. If the states of the observers are not compatible with the communication data, the adjacency matrix will be modified with the correction values applied by the observers. The efficiency of the proposed method has been investigated by using simulations and experimental results. As a suggestion and continuation of the work in this paper, it is recommended that this method be extended for resiliency against stealth attacks. According to the results, it has been shown that by using the proposed method, the DC microgrid network will be resilient against false data injection attacks and the consensus will not deviate.

## Data Availability

Not applicable.
